# Management strategy after diagnosis of Abernethy malformation: a case report

**DOI:** 10.1186/1752-1947-6-167

**Published:** 2012-06-28

**Authors:** Caroline DM Witjes, Jan NM Ijzermans, Anton Vonk Noordegraaf, TC Khe Tran

**Affiliations:** 1 Department of Hepatobiliary and Transplantation Surgery, Erasmus Medical Center, University Medical Center Rotterdam, P.O. Box 2040, Rotterdam, The Netherlands; 2 Department of Pulmonary Medicine, VU University Medical Center Amsterdam, P.O. Box 7057, Amsterdam, MB, 1007, The Netherlands

**Keywords:** hepatocellular carcinoma, arteriovenous malformation, Abernethy malformation, pulmonary hypertension

## Abstract

**Introduction:**

The Abernethy malformation is a rare anomaly with a widely variable clinical presentation. Many diagnostic dilemmas have been reported. Nowadays, with the evolution of medical imaging, diagnosis can be made more easily, but management of patients with an Abernethy malformation is still open for discussion.

**Case presentation:**

In this case study, we describe a 34-year-old Caucasian man who presented with a large hepatocellular carcinoma in the presence of an Abernethy malformation, which was complicated by the development of pulmonary arterial hypertension.

**Conclusion:**

This case underlines the importance of regular examination of patients with an Abernethy malformation, even in older patients, to prevent complications and to detect liver lesions at an early stage.

## Introduction

The Abernethy malformation is an extremely rare anomaly of the splanchnic venous system. It is named after John Abernethy, who first reported the anomaly in 1793. He described a portocaval shunt completely bypassing the liver after a postmortem examination of a 10-month-old girl [1]. Over 80 cases have been described since, mostly in patients ages 18 years and younger [2,3]. The Abernethy malformation is divided into two types. The described cases in the literature often refer to patients with a type I malformation, which is an anomaly defined by an absence of intrahepatic portal veins, thus lacking liver perfusion with portal blood. Type I anomalies may be further divided into subtypes A and B, defined as the superior mesenteric and splenic veins draining separately into the inferior caval vein in type IA and draining from a common trunk in type IB [3]. A type II shunt is defined as a malformation of the portal vein leading to perfusion of the liver via a partial shunt.

Earlier case reports focused on the widely variable forms of clinical presentation and the difficulties encountered in diagnosing the malformation [2]. In recent decades, portosystemic shunts have been recognized more frequently because of the evolution and expansion of medical imaging. Early recognition of a portosystemic shunt is important, given the detrimental effects of shunting on the liver and on the other organs over the long term. In addition, it has been described that liver lesions may develop during childhood. These liver lesions may consist of benign tumors such as focal nodular hyperplasia, hepatocellular adenoma or nodular regenerative hyperplasia. Malignant lesions such as hepatocellular carcinoma (HCC) also can develop, however, and they are most often described in patients with a type I shunt [3-6].

We present the case of a 34-year-old man who was diagnosed with the Abernethy malformation type II three years before a large HCC was treated successfully.

## Case presentation

A 34-year-old Caucasian man was referred to our hospital with complaints of abdominal pain in the right upper quadrant and pain in his right shoulder. The pain was not associated with nausea or vomiting, and there was no history of fever or jaundice.

His medical history included an open ductus venosus and an Abernethy malformation complicated by the development of pulmonary arterial hypertension (mean pulmonary artery pressure 48mmHg), which had been diagnosed three years before and treated successfully with the endothelin receptor antagonist bosentan. At the time of presentation, he was in New York Heart Association functional class I. Physical examination displayed no signs of icterus, and cardiac examination revealed a hyperkinetic cardiac impulse outside the midclavicular line and a loud second heart sound. He had a soft abdomen without organomegaly. His laboratory test showed a hemoglobin level of 9.2mmol/L, an elevated liver enzyme profile with aspartate aminotransferase 46U/L, alanine aminotransferase 50U/L and bilirubin 32μmol/L. α-Fetoprotein (AFP) concentration was normal (3μg/L) as was hepatic synthetic function.

**Figure 1:**
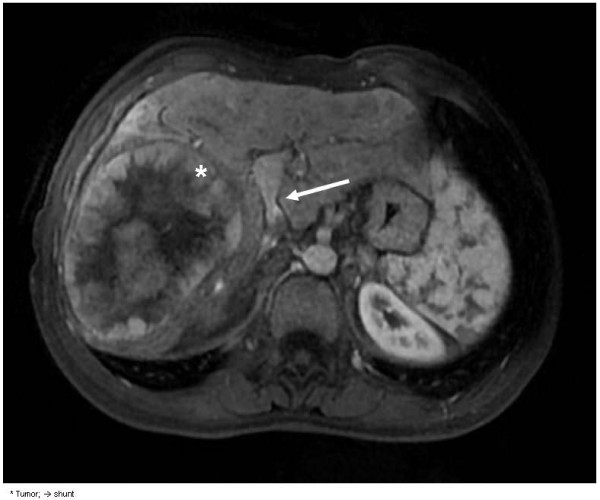
Magnetic resonance angiography showing hepatocellular carcinoma and a portocaval shunt.

**Figure 2:**
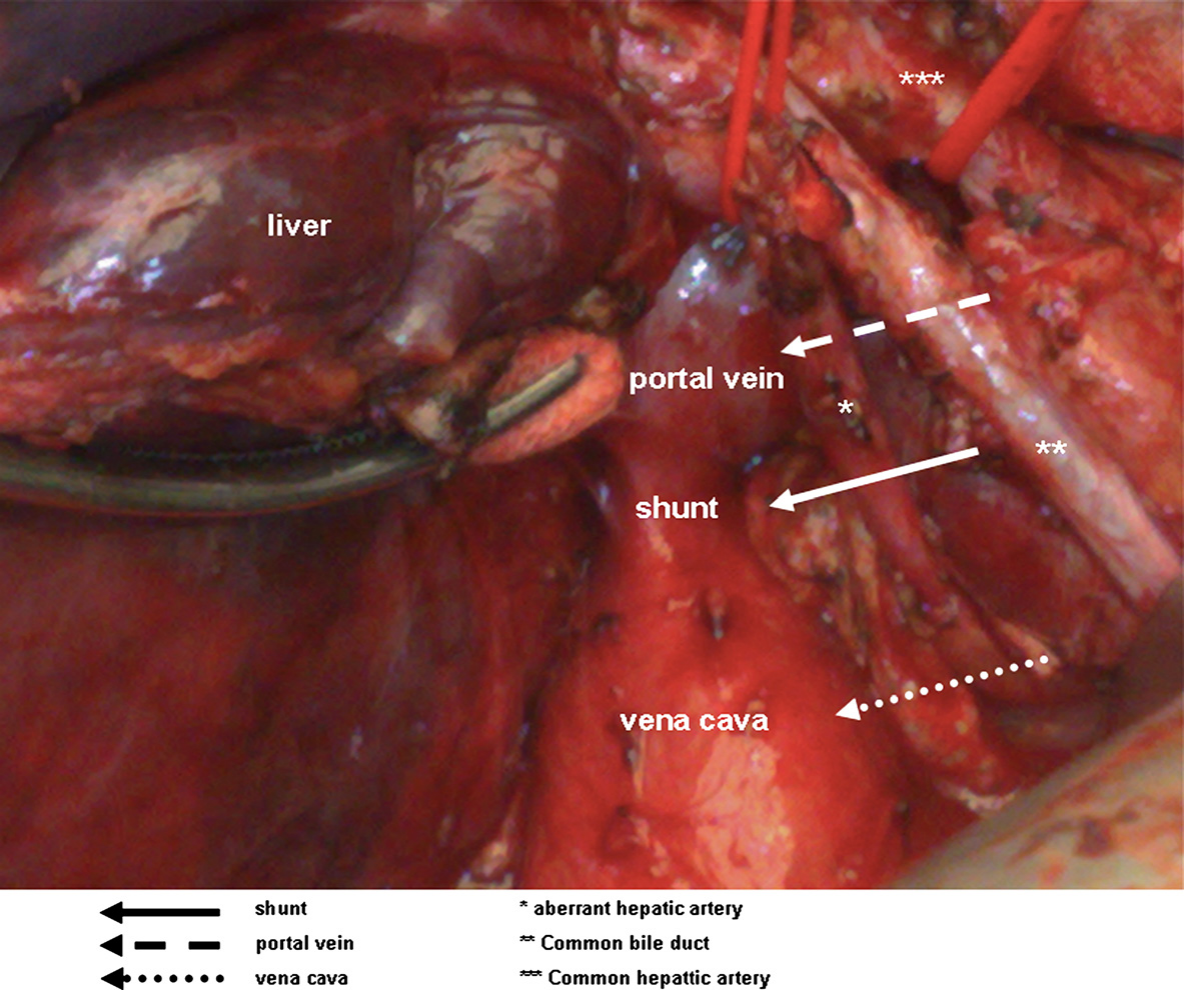
The portocaval shunt peri-operatively.

**Figure 3:**
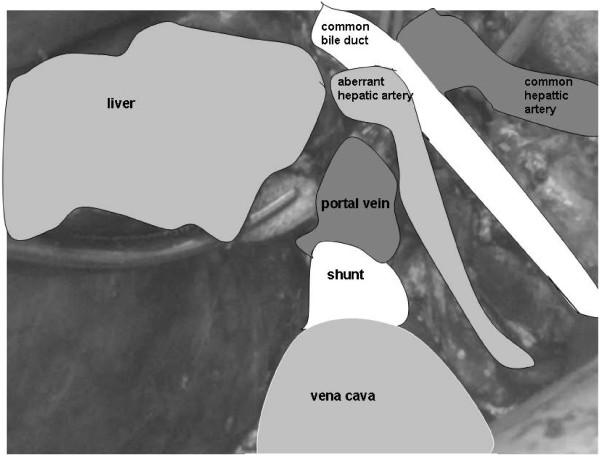
The portocaval shunt peri-operatively.

Computed tomography (CT) displayed the presence of a tumor, and magnetic resonance imaging (MRI) was performed for characterization of the lesion. The lesion was hypointense on T1-weighted images and hyperintense on T2-weighted images in the venous phase, and arterial enhancement and washout were demonstrated, as is typical for a HCC (Figure 1). The presence of a shunt of 2 cm between the left extrahepatic portal vein and the inferior vena cava was confirmed (Figures 2 and 3). Preoperatively, workup included several tests, such as lung capacity (lung function test), cardiac function (electrocardiogram) and hepatic synthetic function (laboratory tests). If the results of these tests were normal, the patient would be eligible for surgery. In the preoperative workup, no biopsy of the tumor neither a biopsy of the normal liver parenchyma was found necessary. Because of his pulmonary hypertension, he was categorized as an American Society of Anesthesiologists (ASA) class 3 patient. There were no other contraindications for surgery, and the patient underwent a successful right hemihepatectomy and surgical discontinuation of the portocaval shunt. Postoperatively, his pulmonary hypertension increased, and he needed oxygen for five days, after which his condition recovered. Furthermore, a small biloma was diagnosed, which was treated by percutaneous drainage.

Histological examination of the resected specimen revealed features of grade II fibrosis [7]. A tumor with a diameter of 16cm was characterized as HCC with the presence of microvascular invasion. A tumor-free surgical resection of 5mm was recorded.

The follow-up of the patient consisted of a routine serum tumor marker AFP determination and contrast-enhanced CT or MRI at six months intervals starting three to six months after surgery. After nearly two years of follow-up, the patient is doing well and is free of HCC recurrence. His cardiac output is normalized, and his pulmonary hypertension is stable.

## Discussion

Treatment of congenital malformations of the portal system depends on the type of portocaval fistulas, the presenting symptoms, complications and comorbidity. Treatment may vary from surgical correction of the shunt to even liver transplantation [8].

Patients with the Abernethy malformation have been described in several case reports, most (80%) of them involving children ages 18 years and younger [2,3]. Management strategies for children with a type I shunt have been developed, with close monitoring of clinical, biochemical and radiological parameters in follow-up being advocated [4]. When an Abernethy malformation is detected, surgical closure of the shunt should be considered. If the shunt cannot be closed, then the patient should be carefully followed.

To date, no management strategy has been described for adult patients with a type II shunt. We propose regular follow-up of patients with a type II shunt as well. Absence of a decent portal circulation and systemic diversion of portal vein flow may have consequences for hepatic development, function and regenerative capacity, thus predisposing such patients to the development of fibrosis, nodular dysplasia or HCC.

Although it has long been considered that essentially all HCCs arise from cirrhotic changes, recent reports have indicated that they can occur in 40% of the patients without cirrhosis of chronic liver disease such as that in our patient [9].

Patients infected with hepatitis B or patients with cirrhosis due to hepatitis C virus infection, alcohol abuse or another cause are patients at risk for the development of HCC. These patients are encountered in a surveillance program. The patients at risk are screened at six to 12 months intervals using liver ultrasonography and serum AFP levels [10]. In cases of abnormal findings, more appropriate follow-up and management can lead to early detection of potentially malignant dysplastic lesions. In our patient, the tumor was large at diagnosis. The patient was thought to have enough functional residual liver tissue, which made a liver resection (hemihepatectomy right) technically possible, although it was risky in this particular case because of the size of the tumor with regard to the radicality of the resection. Liver resections of large malignant tumors have been described before [11]. In cases of a small tumor, patients can be treated with curative intention more often, and five years survival rates over 70% can be achieved [4].

## Conclusion

This case underlines the importance of regular examination of patients with an Abernethy malformation, even in older patients, to prevent complications and to detect liver lesions at an early stage.

## Consent

Written informed consent was obtained from the patient for publication of this case report and any accompanying images. A copy of the written consent is available for review by the Editor-in-Chief of this journal.

## Abbreviation

AFP, α-fetoprotein; CT, computed tomography; HCC, hepatocellular carcinoma.

## Competing interests

The authors declare that they have no competing interests.

## Authors’ contributions

All authors made substantial contributions to this case study’s conception and design and the acquisition, analysis, and interpretation of data (CW, JI, AV and TT). All authors were involved in drafting and revising the manuscript and gave their final approval of the version to be published (CW, JI, AV and TT). Conception and design: CW, JI, AV and TT conceived and designed the study. CW, AV and TT acquired the data. CW, JI and TT analyzed the data. CW, JI, AV and TT interpreted the data. All authors read and approved the final manuscript.
